# Association Between Body Condition Score and Early Radiographic Osteotomy Healing Following Tibial Plateau Leveling Osteotomy in Dogs: A Retrospective Study

**DOI:** 10.3390/ani16203252

**Published:** 2026-10-16

**Authors:** Youngkwang Ryu, Jeonghyun Seo, Kyungmin Song, Chun-Sik Bae

**Affiliations:** 1 Department of Veterinary Surgery, College of Veterinary Medicine, Chonnam National University, Gwangju 61186, Republic of Korea; yyk4510@naver.com; 2 Time Animal Medical Center, 57 Dunsan-Ro, Seo-Gu, Daejeon 35233, Republic of Korea; dvmseo@gmail.com (J.S.); skmrudals@naver.com (K.S.)

**Keywords:** canine, cranial cruciate ligament disease, body condition score, tibial plateau leveling osteotomy, radiographic osteotomy healing, mRUST, tibial plateau angle

## Abstract

Body condition may be associated with postoperative orthopedic outcomes, but its relationship with early radiographic osteotomy healing following tibial plateau leveling osteotomy (TPLO) remains unclear. This retrospective study evaluated the association between preoperative body condition score (BCS) and early radiographic outcomes after TPLO for canine cranial cruciate ligament disease. Seventy-six stifles from 68 dogs with eligible radiographs obtained 42–70 days postoperatively were included. Radiographic osteotomy healing was assessed using a TPLO-specific modified Radiographic Union Scale for Tibial fractures (mRUST), and tibial plateau angle (TPA) was measured preoperatively, immediately postoperatively, and at follow-up. Seventy-one stifles were evaluable for mRUST and 72 for follow-up TPA. Higher BCS was associated with lower mRUST after adjustment for age, body weight, and follow-up interval (β = −0.433 per BCS point; 95% CI, −0.565 to −0.301; *p* < 0.001). Higher BCS was also associated with a modestly greater postoperative TPA change in the prespecified secondary analysis (β = +0.340° per BCS point; 95% CI, 0.021–0.659; *p* = 0.037). Higher preoperative BCS was associated with lower early radiographic osteotomy healing scores and a greater increase in TPA during follow-up after TPLO.

## Simple Summary

A common operation for cruciate ligament disease in dogs involves cutting and rotating the upper part of the shinbone. X-rays are then used to monitor healing of this bone cut. This retrospective study examined whether body condition before surgery was associated with the extent of healing visible on early follow-up X-rays. Seventy-six operated knees were included, with follow-up approximately 6–10 weeks after surgery. Dogs with higher body condition scores had lower radiographic healing scores after accounting for age, body weight, and follow-up timing. Higher body condition scores were also associated with a small increase in the angle of the upper shinbone during follow-up. These findings suggest that body condition may help explain variation in early healing after this operation. Further studies should determine whether these differences affect the time needed for complete healing or the recovery of normal limb function.

## 1. Introduction

Cranial cruciate ligament disease (CCLD) is one of the most common orthopedic disorders affecting the canine stifle and frequently results in pain, instability, and progressive osteoarthritis [1,2,3]. Tibial plateau leveling osteotomy (TPLO) is widely used for surgical stabilization of CCLD and aims to provide dynamic stability by altering the geometry of the proximal tibia [4,5,6]. Following TPLO, radiographic assessment is routinely used to monitor progression of osteotomy healing [7,8].

Radiographic osteotomy healing following TPLO may vary with patient characteristics, local surgical factors, and postoperative management [8,9,10]. Several scoring systems have been proposed to standardize the assessment of healing. A TPLO-specific modified Radiographic Union Scale for Tibial fractures (mRUST) provides a semiquantitative assessment of radiographic osteotomy healing and has demonstrated improved inter-rater reliability compared with subjective assessment [7]. Radiographic positioning can also substantially influence apparent healing scores, emphasizing the importance of standardized image selection and assessment [8].

Body condition score (BCS) is a practical clinical measure used to estimate body condition in dogs and correlates with objective measures of adiposity, including body fat assessed using dual-energy X-ray absorptiometry (DXA) [11,12,13]. Body weight and adiposity have been identified as factors associated with CCLD in clinical studies and reviews of its multifactorial pathogenesis [14,15,16,17]. In dogs, adiposity has also been linked to metabolic changes and altered inflammatory adipokine profiles, providing a biological basis for considering effects beyond absolute body mass [18,19]. In the human orthopedic literature, obesity has been associated with differences in bone mineral density and bone quality [20], as well as longer time to union or increased nonunion risk in clinical fracture studies [21,22,23]. These findings support investigation of body condition as a patient-related factor in postoperative skeletal healing, although its relationship with early radiographic osteotomy healing following TPLO in dogs remains poorly characterized.

The objective of this study was to investigate the association between preoperative BCS and early radiographic osteotomy healing following TPLO in dogs with CCLD. We hypothesized that higher preoperative BCS would be associated with lower early postoperative mRUST scores. Changes in TPA during the same follow-up period were evaluated as a secondary radiographic outcome.

## 2. Materials and Methods

### 2.1. Study Design and Case Selection

This retrospective study reviewed the medical, surgical, and radiographic records of dogs that underwent tibial plateau leveling osteotomy (TPLO) for cranial cruciate ligament disease (CCLD) at a single veterinary referral center between January 2021 and December 2025. TPLO procedures performed during the study period were screened, and only stifles treated with TPLO alone were considered eligible. Stifles that underwent concurrent medial patellar luxation correction or another concurrent orthopedic procedure that could affect interpretation of postoperative osteotomy healing were excluded.

To be included in the primary radiographic cohort, stifles were required to have preoperative and immediate postoperative radiographs and an eligible postoperative radiographic examination obtained between postoperative days (PODs) 42 and 70. When more than one examination was available within this interval, the examination closest to POD 56 was selected; if two examinations were equally distant from POD 56, the earlier examination was selected. Of 103 TPLO-only stifles identified during the study period, 27 were excluded because no eligible radiographic follow-up was available within the predefined interval, leaving 76 stifles from 68 dogs in the primary radiographic cohort.

The stifle was considered the primary unit of analysis. Because both stifles from the same dog could be included, non-independence of observations within dogs was accounted for in the statistical analyses using dog-level clustered standard errors, with an additional dog-level random-intercept model used as a sensitivity analysis.

### 2.2. Clinical Variables and Body Condition Score

For each case, age (recorded and analyzed in months), sex, body weight, preoperative body condition score (BCS), operated limb, surgeon, follow-up interval, and radiographic variables were extracted from the medical record.

Preoperative BCS was obtained from the routine clinical record at the time of preoperative assessment. BCS had been assigned by attending veterinary staff using the established 9-point body condition scoring system described by Laflamme [11]. Assessment was based on visual inspection and palpation, including the ease of rib palpation, visibility of the waist and abdominal tuck, and the extent and distribution of palpable fat deposition. Because this was a retrospective study, BCS assessments had been performed by different members of the veterinary staff rather than by a single standardized observer. For descriptive presentation, stifles were categorized into BCS 4–5, 6–7, and 8–9 groups. For inferential analyses, BCS was retained as a continuous variable. BCS was recorded at the preoperative visit, typically on the day of surgery or the preceding day.

### 2.3. Surgical Procedure and Postoperative Management

All procedures were performed by one of two veterinarians experienced in small-animal orthopedic surgery using a standard TPLO technique [5]. Briefly, a radial osteotomy of the proximal tibia was performed, the tibial plateau segment was rotated to reduce the tibial plateau angle, and the osteotomy was stabilized with a TPLO plate. All osteotomies were stabilized using a single TPLO plate from the same manufacturer (DOIFF Co., Ltd., Suncheon, Republic of Korea). Plate size varied among cases and was selected by the attending surgeon according to patient size. Fixation used a combination of compression and locking screws, without additional plates or pins for definitive fixation. The osteotomy surfaces were closely apposed and compressed to minimize the gap; postoperative osteotomy gap measurements were not available. Because this study spanned 5 years and involved two surgeons, a single uniform target postoperative TPA was not imposed retrospectively for study inclusion.

Postoperative management was generally similar among cases but was not prospectively standardized. Management typically included nonsteroidal anti-inflammatory medication for postoperative analgesia, antimicrobial therapy according to the attending clinician’s protocol, and restriction of physical activity for approximately 2–4 weeks. Activity was subsequently increased gradually according to clinical recovery and follow-up assessment. Return to normal activity was individualized rather than determined by a fixed postoperative time point.

### 2.4. Radiographic Follow-Up and Image Selection

Radiographic examinations consisted of mediolateral and craniocaudal projections. The predefined primary follow-up window was PODs 42–70, centered on an intended 8-week assessment. The examination closest to POD 56 was selected according to the rules described above. When multiple mediolateral projections were available at the selected examination, the best-positioned diagnostic-quality projection was selected for TPA measurement. For mRUST assessment, the complete selected follow-up radiographic set, including the mediolateral and craniocaudal projections, was available to the readers.

Radiographs were reviewed for diagnostic quality, positioning, visualization of the relevant anatomic landmarks, and evaluability of the osteotomy cortices. Because mRUST scoring and TPA measurement require different radiographic features, evaluability for these two outcomes was assessed independently.

### 2.5. Tibial Plateau Angle Measurement

Tibial plateau angle (TPA) measurements were performed by a single investigator (Y.R.) using the angle measurement tools in INFINITT PACS (INFINITT Healthcare Co., Ltd., Seoul, Republic of Korea). TPA was measured on mediolateral radiographs using standardized anatomical landmarks [24]. The tibial mechanical axis was defined as the line connecting the center of the intercondylar eminences and the center of the talus. The tibial plateau line was defined by the cranial and caudal extents of the medial tibial plateau, and TPA was defined as the acute angle between the tibial plateau line and a line perpendicular to the tibial mechanical axis. Because limb positioning can materially affect radiographically measured TPA, only images in which the required landmarks could be identified reliably were used [24,25].

TPA was measured preoperatively, immediately postoperatively, and at the selected primary follow-up examination. Each TPA was measured twice by the same investigator approximately 3 weeks apart, and the arithmetic mean of the two measurements was used for statistical analysis. Postoperative TPA change was calculated as ΔTPA = follow-up TPA—immediate postoperative TPA. Follow-up radiographs were excluded from the ΔTPA analysis when positioning or image quality prevented reliable identification of the landmarks required for TPA measurement. Intraobserver reliability of the repeated TPA measurements was evaluated separately for the preoperative, immediate postoperative, and follow-up measurements. Repeated measurements and reliability assessment were used to characterize measurement variability, which has been documented for radiographic TPA assessment [26]. The investigator was blinded to BCS during both measurement sessions.

### 2.6. mRUST Assessment of Osteotomy Healing

Early radiographic osteotomy healing was assessed at the selected primary follow-up examination using the TPLO-specific modified Radiographic Union Scale for Tibial fractures (mRUST) described by Walker et al. [7]. Three cortices—cranial, caudal, and lateral—were evaluated. The medial cortex was omitted because its visualization may be obscured by superimposition of the TPLO plate. Each evaluable cortex was assigned a score from 1 to 4: 1 indicated no visible callus; 2 indicated callus formation without cortical bridging; 3 indicated bridging callus with the osteotomy line still visible; and 4 indicated advanced bridging/remodeling with the osteotomy line no longer visible. The three cortical scores were summed to produce a total TPLO-specific mRUST score ranging from 3 to 12, with higher scores indicating more advanced radiographic healing.

Before scoring, follow-up image sets were anonymized and assigned randomized identifiers. Two veterinarians independently evaluated the radiographic examinations and were blinded to case identity, BCS, body weight, surgeon, TPA measurements, and other clinical information. The mean of the two readers’ total mRUST scores was used as the primary radiographic healing outcome. Interobserver reliability of the total mRUST scores was evaluated using an intraclass correlation coefficient.

### 2.7. Radiographic Quality Control and Evaluability

Radiographic evaluability was assessed independently for mRUST scoring and TPA measurement because the two outcomes required different anatomical and positioning criteria [8,24,25]. For mRUST, the cranial, caudal, and lateral cortices had to be sufficiently visualized to permit reliable scoring by both readers. Five stifles were excluded because implant/plate superimposition prevented reliable assessment of the required cortices: one image set was excluded before blinded scoring, and four additional image sets did not yield complete three-cortex scores from both readers. Consequently, 71 stifles were included in the primary mRUST analysis.

For TPA, follow-up radiographs were excluded when positioning or landmark visualization prevented reliable angle measurement. Four stifles were excluded on this basis, leaving 72 stifles for follow-up TPA and ΔTPA analyses. These four stifles did not overlap with the five stifles excluded from the primary mRUST analysis.

### 2.8. Outcome Measures

The primary outcome was the association between preoperative BCS and early radiographic osteotomy healing, quantified using the mean of the two readers’ total mRUST scores at the primary follow-up examination. The secondary radiographic outcome was postoperative change in TPA (ΔTPA) between the immediate postoperative and primary follow-up examinations. Measurement reliability outcomes were interobserver reliability for mRUST scoring and intraobserver reliability for repeated TPA measurements.

### 2.9. Statistical Analysis

Continuous variables were summarized using mean ± standard deviation (SD) and, where informative, median and interquartile range (IQR). Categorical variables were summarized as frequencies and percentages. Baseline characteristics were presented descriptively across BCS groups without separate baseline significance testing. Baseline characteristics of included and excluded TPLO-only stifles were also compared descriptively. Breed distribution was summarized at the dog level to avoid counting bilateral cases twice.

The primary association between preoperative BCS and mean mRUST score was evaluated using linear regression with BCS entered as a continuous predictor. An unadjusted model was fitted first, followed by an adjusted model including age (months), body weight, and follow-up interval as prespecified clinically relevant covariates. Because more than one stifle from the same dog could be included, dog-clustered sandwich-robust standard errors were used to account for within-dog correlation. Confidence intervals and *p* values were calculated using the large-sample normal approximation. Model diagnostics included residual-versus-fitted, normal Q–Q, scale-location, and BCS partial-residual plots; a centered quadratic BCS term assessed departure from linearity. Residual normality was explored using the Shapiro–Wilk test. Leverage, Cook’s distance, and leave-one-dog-out analyses were used to assess influential observations.

Sensitivity analyses used a linear mixed-effects model with a dog-level random intercept and reader-specific proportional-odds ordinal regression models fitted to each reader’s integer mRUST scores. Both sensitivity analyses included the same covariates as the primary analysis: age (months), body weight, and follow-up interval. Dog-clustered robust covariance was used for the ordinal regression models. The association between BCS and ΔTPA was evaluated as a secondary analysis using an adjusted linear model with the same covariates and dog-clustered robust standard errors. Exploratory sensitivity analyses additionally adjusted the ΔTPA model for immediate postoperative TPA and the primary mRUST model for breed group (Maltese, Bichon Frise, Poodle, or other breeds). Breed groups were used because individual breed counts were small. Mixed-model confidence intervals and *p* values used a normal Wald approximation conditional on the fitted variance parameters.

Interobserver reliability of mRUST scoring was quantified using a two-way random-effects, absolute-agreement, single-measure intraclass correlation coefficient [ICC(2,1)] with a 95% confidence interval. Intraobserver reliability of TPA measurements repeated by the same investigator was evaluated using a two-way mixed-effects, absolute-agreement, single-measure intraclass correlation coefficient [ICC(A,1)] with a 95% confidence interval, separately for the preoperative, immediate postoperative, and follow-up measurements. Absolute repeatability was described using the mean absolute difference between the two measurements and descriptive 95% limits of agreement, calculated as the mean signed difference (second minus first measurement) ± 1.96 SD of the differences. These summaries were calculated for each TPA time point and for independently calculated ΔTPA values from the two measurement sessions. They characterize repeated readings of the same images, not between-examination positioning error.

Generative AI assistance was used during manuscript preparation and statistical calculations and verification, as disclosed in the Acknowledgments. It was not used to assign clinical BCS values, measure TPA, or score radiographic osteotomy healing.

Statistical analyses were performed using Python version 3.12.14 with NumPy version 2.3.5 and SciPy version 1.17.0. Custom Python scripts implemented linear regression with dog-clustered sandwich-robust covariance estimation, proportional-odds ordinal regression, a Gaussian random-intercept model fitted by maximum likelihood, and intraclass correlation coefficients with confidence intervals. All tests were two-sided, and *p* < 0.05 was considered statistically significant. The reproducible analysis script and input specification are provided as Supplementary Analysis Code.

## 3. Results

### 3.1. Study Population

**Figure 1:**
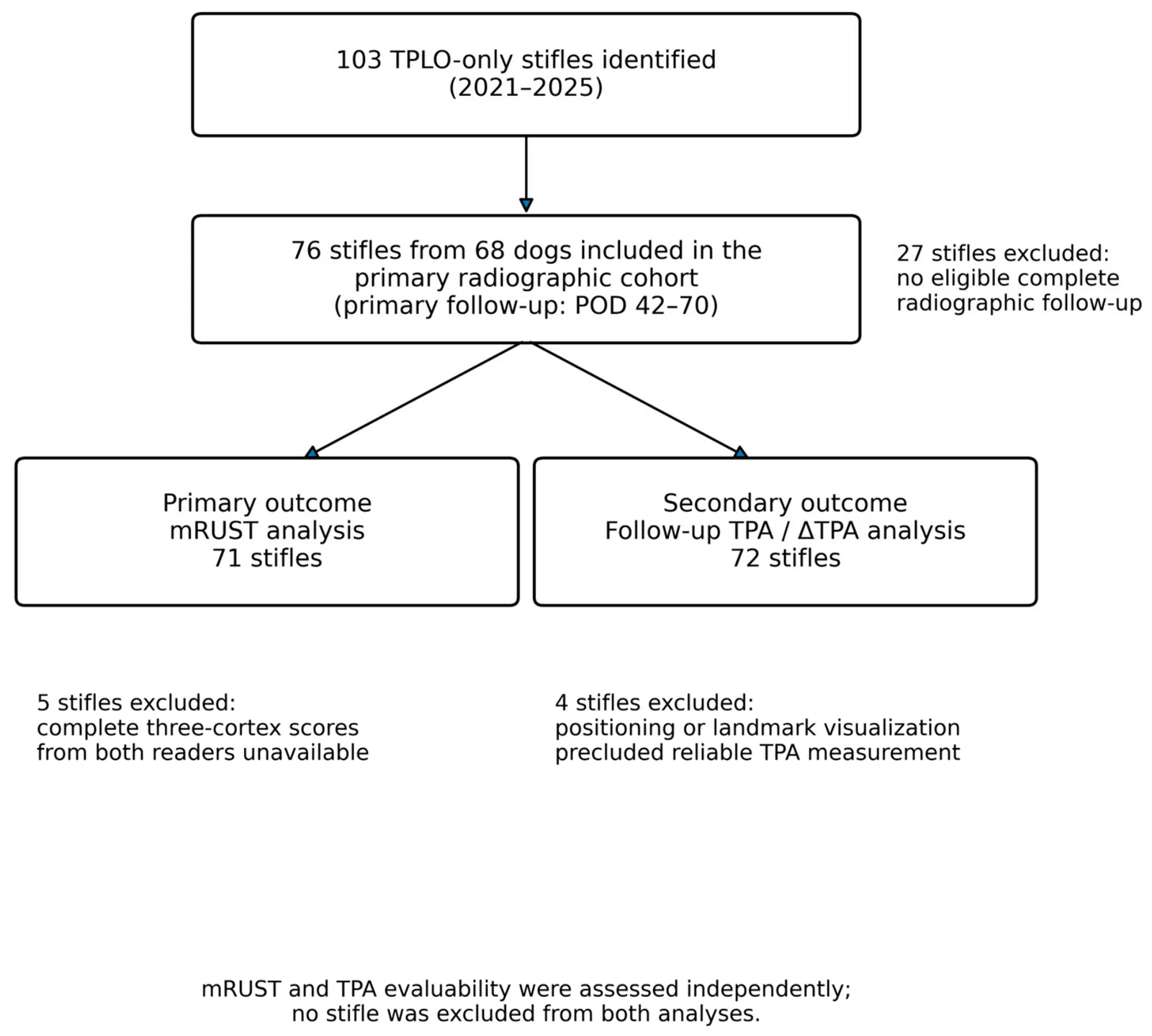
Flow diagram of case selection and radiographic outcome evaluability. Of 103 TPLO-only stifles identified between 2021 and 2025, 27 were excluded because no eligible radiographic follow-up was available within PODs 42–70, leaving 76 stifles from 68 dogs in the primary radiographic cohort. Complete mRUST scores from both blinded readers were available for 71 stifles. Follow-up TPA and ΔTPA were evaluable in 72 stifles. Radiographic evaluability for mRUST and TPA was assessed independently, and the excluded stifles did not overlap. POD, postoperative day; TPA, tibial plateau angle; TPLO, tibial plateau leveling osteotomy.

A total of 103 TPLO-only stifles were identified during the study period. Of these, 76 stifles from 68 dogs had an eligible primary follow-up radiographic examination between PODs 42 and 70 and were included in the primary radiographic cohort. The mean follow-up interval was 53.03 ± 6.86 days. The cohort included 21 stifles in the BCS 4–5 group, 37 in the BCS 6–7 group, and 18 in the BCS 8–9 group. The mean age and body weight of the overall cohort were 96.33 ± 36.51 months and 8.03 ± 5.46 kg, respectively. Baseline characteristics are summarized in Table 1. The study flow and outcome-specific radiographic evaluability are summarized in Figure 1. The excluded stifles had a similar descriptive BCS distribution to the included stifles (mean ± SD, 6.41 ± 1.45 versus 6.46 ± 1.33; Table S1). All 68 included dogs were neutered (44 males and 24 females). The most frequent breeds were Maltese (15 dogs), Bichon Frise (13), and Poodle (11); the complete distribution is provided in Table S3. No implant failure was documented in the included cases, and no revision surgery was performed because of osteotomy fragment displacement or nonunion.

### 3.2. Radiographic Osteotomy Healing

Five stifles were excluded from the primary mRUST analysis because complete three-cortex scores from both blinded readers were unavailable, leaving 71 stifles for analysis. The overall mean mRUST score was 8.54 ± 1.10. Mean mRUST scores were 9.22 ± 0.99 in the BCS 4–5 group, 8.53 ± 1.00 in the BCS 6–7 group, and 7.86 ± 1.03 in the BCS 8–9 group (Table 2). The corresponding median [IQR] values were 9.25 [8.50–10.00], 8.00 [8.00–9.00], and 8.00 [7.13–8.38], respectively.

### 3.3. Body Condition Score and Radiographic Osteotomy Healing

**Figure 2:**
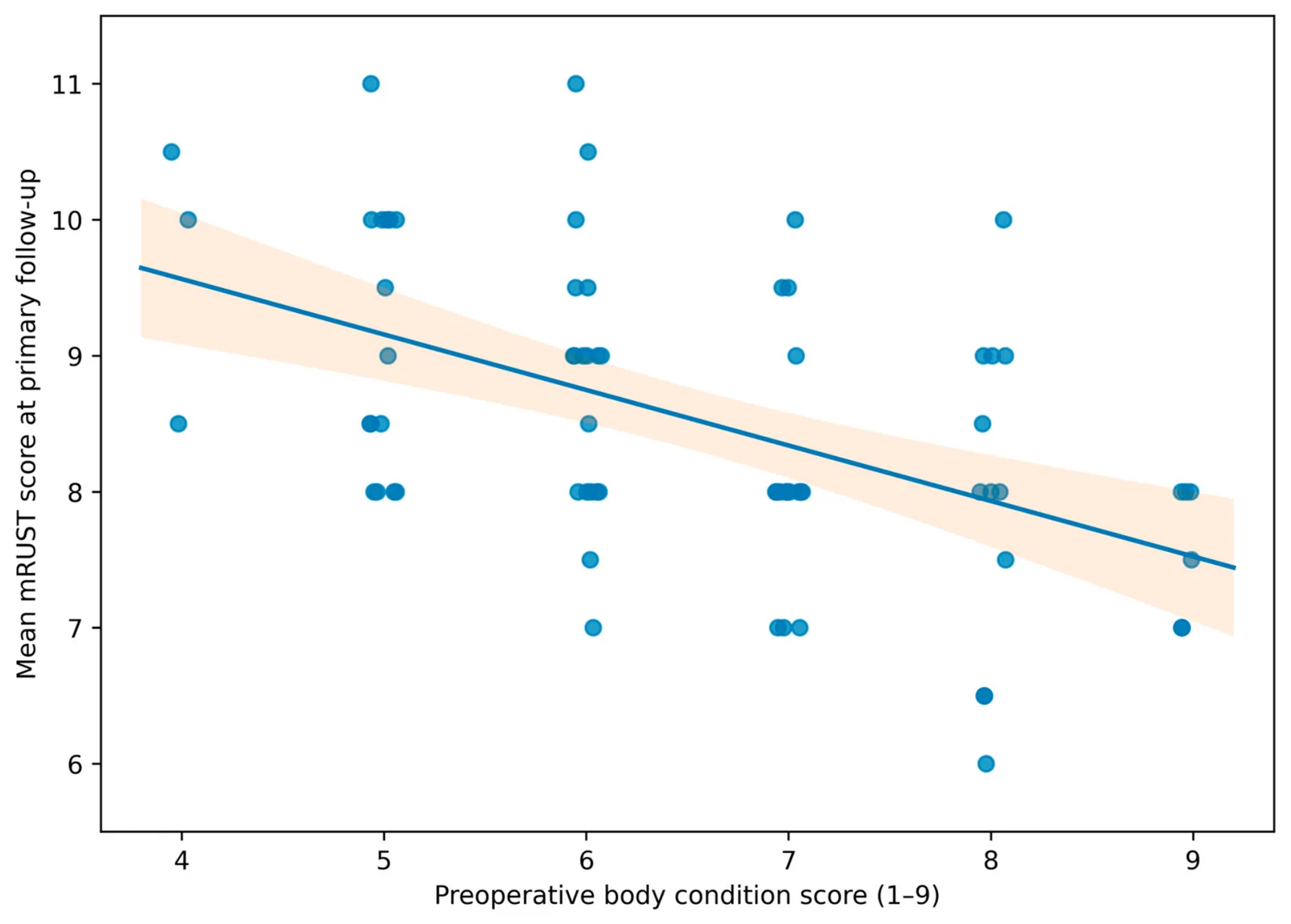
Association between preoperative body condition score (BCS) and early radiographic osteotomy healing following tibial plateau leveling osteotomy. Individual points represent the mean mRUST score of two blinded readers for each stifle at the primary follow-up examination (*n* = 71); points were horizontally jittered to reduce overlap. The blue points represent individual stifles, the blue line shows the fitted unadjusted linear regression, and the pale orange shaded band represents the 95% confidence interval for the fitted mean. In the adjusted analysis, higher BCS was associated with lower mean mRUST after adjustment for age, body weight, and follow-up interval (β = −0.433 per BCS point; 95% CI, −0.565 to −0.301; *p* < 0.001). mRUST, modified Radiographic Union Scale for Tibial fractures.

In the unadjusted analysis, higher preoperative BCS was associated with a lower mean mRUST score (β = −0.408 per 1-point increase in BCS; 95% CI, −0.526 to −0.290; *p* < 0.001). After adjustment for age, body weight, and follow-up interval, the association remained significant (β = −0.433; 95% CI, −0.565 to −0.301; *p* < 0.001) (Table 3). The linear mixed-effects sensitivity analysis yielded the same point estimate (β = −0.433; 95% CI, −0.603 to −0.263; *p* < 0.001); the estimated dog-level random-intercept variance was approximately zero, indicating little additional between-dog variance after accounting for the fixed effects. Reader-specific ordinal sensitivity analyses showed associations in the same direction (Reader 1: OR, 0.492; 95% CI, 0.359–0.675; *p* < 0.001; Reader 2: OR, 0.434; 95% CI, 0.306–0.617; *p* < 0.001). The individual-stifle distribution and unadjusted visual trend are shown in Figure 2. An exploratory model additionally adjusting for breed group yielded a consistent association (β = −0.483; 95% CI, −0.635 to −0.332; *p* < 0.001; Table S4). Diagnostic plots are provided in Figure S1. The quadratic BCS term did not provide evidence of nonlinearity (*p* = 0.486), and the residual Shapiro–Wilk test yielded *p* = 0.567. Fitted values remained within the mRUST scale (7.38–9.53). Although four observations exceeded the Cook’s distance screening value of 4/*n*, leave-one-dog-out BCS coefficients ranged from −0.456 to −0.414.

### 3.4. Tibial Plateau Angle Changes

Immediate postoperative TPA was available for all 76 stifles, with a mean value of 4.19 ± 2.43°. Four stifles were excluded from follow-up TPA analysis because radiographic positioning or landmark visualization precluded reliable measurement, leaving 72 stifles for follow-up TPA and ΔTPA analyses. Mean follow-up TPA was 5.42 ± 3.09°, and mean ΔTPA was +1.32 ± 1.92° (range, −1.90° to +8.75°). Although the average increase was small, some cases showed larger increases. Mean ΔTPA values were +1.03 ± 1.35°, +1.17 ± 2.21°, and +1.97 ± 1.75° in the BCS 4–5, 6–7, and 8–9 groups, respectively (Table 2). In the adjusted secondary analysis, higher BCS was associated with a greater postoperative increase in TPA (β = +0.340° per BCS point; 95% CI, 0.021–0.659; *p* = 0.037) (Table 3). Additional adjustment for immediate postoperative TPA yielded a similar BCS coefficient (β = +0.345°; 95% CI, 0.011–0.679; *p* = 0.043; Table S4).

### 3.5. Measurement Reliability

Interobserver reliability for mRUST scoring was good (ICC = 0.840; 95% CI, 0.756–0.897). Intraobserver reliability of repeated TPA measurements was excellent for the preoperative (ICC = 0.965; 95% CI, 0.946–0.978), immediate postoperative (ICC = 0.993; 95% CI, 0.989–0.995), and follow-up measurements (ICC = 0.992; 95% CI, 0.987–0.995). Mean absolute differences between repeated TPA readings were 0.96° preoperatively, 0.23° immediately postoperatively, and 0.31° at follow-up. For ΔTPA, the mean absolute difference was 0.38°, with descriptive 95% limits of agreement of −0.88° to +1.04° (Table S2).

## 4. Discussion

### 4.1. Principal Findings

Higher preoperative BCS was associated with lower early radiographic osteotomy healing scores following TPLO. Each 1-point increase in BCS was associated with an approximately 0.43-point decrease in mean mRUST score, and this association persisted after adjustment for age, body weight, and follow-up interval. The direction of the association was consistent in the linear mixed-effects model and reader-specific ordinal regression analyses. These findings provide a basis for considering body condition alongside absolute body weight when interpreting variation in early radiographic osteotomy healing after TPLO.

### 4.2. Body Condition and Radiographic Osteotomy Healing

BCS correlates with objective estimates of canine adiposity and therefore provides information that complements body weight [12,13]. In human clinical studies, obesity has been associated with longer fracture union times and increased nonunion risk [21,22,23]. Together with evidence of differences in bone quality [20], these findings provide a comparative context for the association observed in the present study. The persistence of the BCS–mRUST association after adjustment for body weight suggests that differences in absolute body mass alone may not fully explain variation in early radiographic healing.

Potential explanations include differences in mechanical loading and the metabolic or inflammatory effects of adipose tissue. In obese dogs, weight loss has been associated with improved insulin sensitivity and reductions in several inflammatory markers [18], while adipokines provide a plausible link between adiposity and systemic metabolic function [19]. In a diet-induced obesity mouse model with accompanying hyperglycemia and insulin resistance, impaired fracture healing was associated with altered early innate and later adaptive immune responses [27]. A separate high-fat-diet mouse study found no impairment of healing in stably fixed femoral fractures [28]. These experimental findings suggest that the relationship between obesity and skeletal repair depends on the metabolic phenotype and healing environment.

Associations between adiposity, body weight, and CCLD have also been reported in dogs [14,15]. Objective body-composition assessment identified greater adiposity in dogs with CCLD than in controls [14], while epidemiologic research found that increasing body weight within breeds was associated with higher odds of CCLD diagnosis [15]. The present study extends this clinical context to an association between preoperative BCS and early radiographic osteotomy healing after TPLO.

### 4.3. Postoperative Tibial Plateau Angle Change

Higher BCS was associated with a greater postoperative increase in TPA in the secondary analysis. The mean increase in the present study (+1.32°) was comparable to the mean increase of 1.5° reported by Moeller et al. at a mean follow-up of 46 days after TPLO [29]. In a retrospective study of small-breed dogs, Cosenza et al. reported mean TPA values of 5.8° immediately postoperatively and 7.3° at 6–8 weeks [30]. TPA changes have also been reported over longer follow-up periods, with their magnitude varying across study populations and follow-up intervals [31,32]. In a recent study restricted to small-breed dogs, long-term TPA change was relatively small and was not associated with body weight [32]. The early association with BCS in the present study adds a patient-related observation to this literature; its clinical relevance requires assessment alongside functional outcomes. The BCS coefficient reflects a group-level association rather than a threshold for clinically meaningful change in an individual dog. The mean absolute difference between repeated ΔTPA readings was 0.38°, with limits of agreement of −0.88° to +1.04°. Small individual changes may therefore be difficult to distinguish from reading variability. These within-image estimates do not capture differences in positioning between postoperative and follow-up examinations [24,25,26]. Larger increases occurred in some cases, but functional outcomes and subtle osteotomy fragment displacement were not systematically assessed; the clinical significance remains uncertain.

### 4.4. Limitations

This retrospective, single-institution study has several limitations. Although the adjusted model included age, body weight, and follow-up interval, postoperative activity, rehabilitation [33], diet [10], and other clinical or surgical factors were not comprehensively characterized. Local surgical factors, including the immediate postoperative osteotomy gap, have been associated with radiographic osteotomy healing scores after TPLO [9]. Residual confounding may therefore remain, and the observed association does not establish a causal effect of BCS. Breed was considered only through broad groups in an exploratory sensitivity analysis, which cannot fully account for individual breed-related differences.

Preoperative BCS values were obtained from routine clinical records and had been assigned by different veterinary staff members. Although the established 9-point scoring system was used, interobserver consistency could not be verified retrospectively. BCS is also semiquantitative and provides limited information about actual body fat mass and distribution. Body composition and metabolic or inflammatory markers were not measured, preventing assessment of the biological mechanisms underlying the association. Exclusion of 27 of 103 TPLO-only stifles because follow-up radiographs were unavailable within PODs 42–70 may have introduced selection bias. Although included and excluded stifles had similar descriptive BCS distributions (Table S1), measured similarities do not exclude differences in unrecorded characteristics, recovery, or follow-up attendance. The findings may therefore not represent all dogs undergoing TPLO.

The study assessed radiographic findings at 6–10 weeks after surgery and did not establish time to final union, functional recovery, or long-term clinical outcomes. Lower early mRUST scores should therefore not be equated with delayed union or nonunion as defined in the human fracture studies [21,22,23]. The human and mouse evidence also involves different species, metabolic phenotypes, and fracture environments from canine TPLO. The secondary BCS–ΔTPA association was modest and its functional significance was not assessed. These limits should be considered when applying the findings to other patient populations or postoperative management settings. Delayed union was not systematically classified during follow-up.

### 4.5. Clinical Interpretation and Future Directions

Preoperative BCS can be considered alongside body weight when interpreting variation in early radiographic osteotomy healing after TPLO. Its availability during routine clinical examinations makes it a practical patient characteristic for preoperative assessment and follow-up interpretation. Prospective studies should combine objective body-composition measurements with standardized serial radiographs and functional assessments to determine whether the observed score differences correspond to longer time to union or differences in clinical recovery. The effects of body-condition management on postoperative healing can then be evaluated in interventional studies.

## 5. Conclusions

Higher preoperative BCS was associated with lower early radiographic osteotomy healing scores following TPLO after adjustment for age, body weight, and follow-up interval. Higher BCS was also associated with a modestly greater postoperative TPA increase in the secondary analysis. These findings support consideration of body condition alongside body weight when interpreting early radiographic outcomes and warrant prospective evaluation using direct body-composition measurements, longitudinal healing assessments, and functional outcomes.

## Figures and Tables

**Table 1:** Baseline characteristics of the primary radiographic cohort.

Characteristic	Overall (*n* = 76)	BCS 4–5 (*n* = 21)	BCS 6–7 (*n* = 37)	BCS 8–9 (*n* = 18)
Age (months), mean ± SD	96.33 ± 36.51	110.62 ± 40.43	89.24 ± 33.54	94.22 ± 34.78
Body weight (kg), mean ± SD	8.03 ± 5.46	6.39 ± 3.95	8.41 ± 6.85	9.16 ± 2.95
Sex, MN/FS, *n*	49/27	15/6	24/13	10/8
Operated limb, L/R, *n*	34/42	10/11	16/21	8/10
Surgeon, A/B, *n*	53/23	12/9	28/9	13/5
Primary FU (POD), mean ± SD	53.03 ± 6.86	53.19 ± 5.48	53.35 ± 7.61	52.17 ± 6.96
Preoperative TPA (°), mean ± SD	28.61 ± 4.42	27.06 ± 3.92	28.68 ± 4.67	30.28 ± 3.98

BCS groups are presented descriptively; BCS was modeled as a continuous predictor in inferential analyses. BCS, body condition score; FS, female spayed; FU, follow-up; L, left; MN, male neutered; POD, postoperative day; R, right; SD, standard deviation; TPA, tibial plateau angle.

**Table 2:** Radiographic outcomes according to preoperative BCS group.

Outcome	Overall	BCS 4–5	BCS 6–7	BCS 8–9
Mean mRUST, mean ± SD	8.54 ± 1.10 (*n* = 71)	9.22 ± 0.99 (*n* = 18)	8.53 ± 1.00 (*n* = 35)	7.86 ± 1.03 (*n* = 18)
Mean mRUST, median [IQR]	8.00 [8.00–9.25]	9.25 [8.50–10.00]	8.00 [8.00–9.00]	8.00 [7.13–8.38]
Immediate postoperative TPA (°), mean ± SD	4.19 ± 2.43 (*n* = 76)	3.51 ± 2.29 (*n* = 21)	4.18 ± 2.34 (*n* = 37)	4.99 ± 2.67 (*n* = 18)
Follow-up TPA (°), mean ± SD	5.42 ± 3.09 (*n* = 72)	4.44 ± 2.97 (*n* = 19)	5.28 ± 2.95 (*n* = 36)	6.82 ± 3.20 (*n* = 17)
ΔTPA, FU—postop (°), mean ± SD	1.32 ± 1.92 (*n* = 72)	1.03 ± 1.35 (*n* = 19)	1.17 ± 2.21 (*n* = 36)	1.97 ± 1.75 (*n* = 17)

The primary mRUST analysis included 71 stifles with complete scores from both blinded readers. TPA: immediate postoperative *n* = 76; follow-up/ΔTPA *n* = 72. ΔTPA = follow-up TPA—immediate postoperative TPA. IQR, interquartile range; mRUST, modified Radiographic Union Scale for Tibial fractures; SD, standard deviation; TPA, tibial plateau angle.

**Table 3:** Associations between preoperative BCS and radiographic outcomes.

Analysis	Outcome	Effect per BCS Point	95% CI	*p* Value
Primary analysis	Mean mRUST	β = −0.433 points	−0.565 to −0.301	<0.001
Mixed-effects sensitivity	Mean mRUST	β = −0.433 points	−0.603 to −0.263	<0.001
Sensitivity analysis—Reader 1	Ordinal mRUST	OR = 0.492	0.359 to 0.675	<0.001
Sensitivity analysis—Reader 2	Ordinal mRUST	OR = 0.434	0.306 to 0.617	<0.001
Secondary analysis	ΔTPA	β = +0.340°	0.021 to 0.659	0.037

All models were adjusted for age (months), body weight, and follow-up interval. Linear and ordinal models used dog-clustered robust covariance and normal-approximation confidence intervals and *p* values. The mixed-effects model included a dog-level random intercept; its variance estimate was zero, and the reported Wald interval and *p* value are conditional on the fitted variance parameters. β represents outcome change per 1-point increase in BCS; OR < 1 indicates lower odds of a higher mRUST category. BCS, body condition score; CI, confidence interval; mRUST, modified Radiographic Union Scale for Tibial fractures; OR, odds ratio; TPA, tibial plateau angle.

## Data Availability

The de-identified data presented in this study are available on request from the corresponding author. The data are not publicly available because they are derived from clinical records of client-owned animals and are subject to owner confidentiality and institutional requirements.

## Supplementary Materials

The following supporting information can be downloaded at https://www.mdpi.com/article/10.3390/ani16203252/s1. Table S1: Baseline characteristics of included and excluded stifles; Table S2: Repeatability of TPA measurements; Table S3: Breed distribution of included dogs; Table S4: Exploratory sensitivity analyses; Figure S1: Diagnostics of the primary linear regression model; Supplementary Analysis Code: Reproducible Python script and input specification.

## Abbreviations

BCS	Body condition score
CCL	Cranial cruciate ligament
CCLD	Cranial cruciate ligament disease
CI	Confidence interval
CrCd	Craniocaudal
FU	Follow-up
ICC	Intraclass correlation coefficient
mRUST	Modified Radiographic Union Scale for Tibial fractures
OR	Odds ratio
POD	Postoperative day
SD	Standard deviation
TPA	Tibial plateau angle
TPLO	Tibial plateau leveling osteotomy
