# Supplementary material for: Association Between Body Condition Score and Early Radiographic Osteotomy Healing Following Tibial Plateau Leveling Osteotomy in Dogs: A Retrospective Study
Source: Animals (Basel). 2026 Oct 16;16(20):3252. doi: 10.3390/ani16203252 (PMC13700849; doi:10.3390/ani16203252)
Supplement: Supplementary file 1 [file animals-16-03252-s001.zip › Supplementary_Materials.pdf]

Supplementary Materials

Association between Body Condition Score and Early Radiographic Osteotomy Healing Following Tibial Plateau Leveling Osteotomy in Dogs: A Retrospective Study

Table S1 Baseline characteristics of included and excluded stifles

Characteristic	Included (n = 76)	Excluded (n = 27)
Age (months), mean ± SD	96.33 ± 36.51	104.04 ± 34.69
Body weight (kg), mean ± SD	8.03 ± 5.46	8.67 ± 3.49
BCS, mean ± SD	6.46 ± 1.33	6.41 ± 1.45
BCS, median [IQR]	6 [5–7]	6 [5–7]
BCS 4–5, n (%)	21 (27.6)	8 (29.6)
BCS 6–7, n (%)	37 (48.7)	13 (48.1)
BCS 8–9, n (%)	18 (23.7)	6 (22.2)

Values are summarized at the stifle level without significance testing. The included and excluded sets comprised 68 and 25 dogs, respectively; one dog contributed a stifle to each set. Exclusion was due to unavailable eligible follow-up radiographs within POD 42–70. BCS, body condition score; IQR, interquartile range; SD, standard deviation.

Table S2 Repeatability of TPA measurements

Measurement	n	Mean absolute difference (°)	Mean signed difference (°)	95% limits of agreement (°)
Preoperative TPA	76	0.96	+0.20	-2.08 to +2.48
Immediate postoperative TPA	76	0.23	+0.02	-0.55 to +0.59
Follow-up TPA	72	0.31	+0.11	-0.64 to +0.86
ΔTPA	72	0.38	+0.08	-0.88 to +1.04

The same investigator measured each image twice approximately 3 weeks apart. Signed differences are second minus first readings. Limits of agreement are descriptive mean differences ± 1.96 SD, without confidence intervals or adjustment for bilateral clustering. ΔTPA was calculated separately within each session. These estimates concern within-image reading repeatability and do not quantify positioning variability between examinations.

Table S3 Breed distribution of included dogs

Breed	Dogs, n	Percentage
Maltese	15	22.1
Bichon Frise	13	19.1
Poodle	11	16.2
Mixed breed	7	10.3
Welsh Corgi	6	8.8
Pomeranian	4	5.9
Spitz	3	4.4
Maltipoo	2	2.9
Chihuahua	1	1.5
Jindo	1	1.5
Labrador Retriever	1	1.5
Cocker Spaniel	1	1.5
Shih Tzu	1	1.5
Yorkshire Terrier	1	1.5
French Bulldog	1	1.5

Each dog is counted once (n = 68); bilateral stifles are not counted twice. Breed labels follow clinical records without inferring a breed subtype.

Table S4 Exploratory sensitivity analyses

Outcome and additional covariate	Stifles / dogs	BCS coefficient	95% CI	p value
Mean mRUST, breed group	71 / 63	−0.483	−0.635 to −0.332	<0.001
ΔTPA, immediate postoperative TPA	72 / 66	+0.345°	0.011 to 0.679	0.043

Both models also included age, body weight, and follow-up interval, with dog-clustered robust covariance. Breed was grouped as Maltese, Bichon Frise, Poodle, or other breeds. These analyses were exploratory additions during revision. The immediate postoperative TPA coefficient was −0.017 (95% CI, −0.163 to 0.129; p = 0.819).

Figure S1 Diagnostics of the primary linear regression model

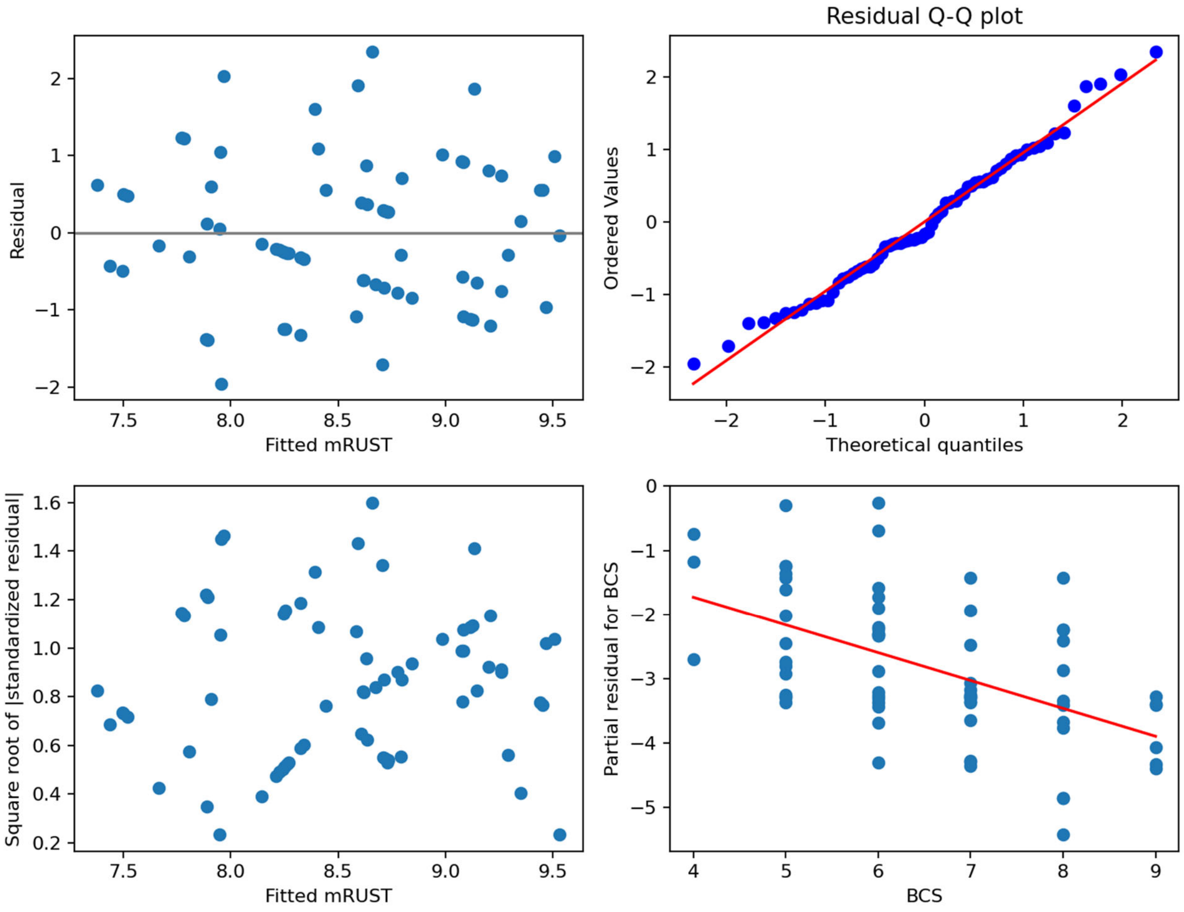

Residual-versus-fitted plot, normal Q–Q plot, scale-location plot, and partial residual plot for BCS (71 stifles). The quadratic BCS term yielded $p = 0.486$; the residual Shapiro–Wilk test yielded $p = 0.567$. Maximum leverage was 0.657 and maximum Cook's distance was 0.160. Four observations exceeded 4/n; leave-one-dog-out BCS coefficients ranged from −0.456 to −0.414. Diagnostics were used to explore model adequacy rather than prove assumptions.
